# Intermittent treatment interruption and its effect on multidrug resistant tuberculosis treatment outcome in Ethiopia

**DOI:** 10.1038/s41598-019-56553-1

**Published:** 2019-12-27

**Authors:** Habteyes H. Tola, Kourosh Holakouie-Naieni, Mohammad A. Mansournia, Mehdi Yaseri, Ephrem Tesfaye, Zemedu Mahamed, Million Molla Sisay

**Affiliations:** 1 Tehran University of Medical Sciences, School of Public Health, Department of Epidemiology and Biostatistics, Tehran, Iran; 2 Ethiopian Public Health Institute, Tuberculosis/HIV Research Directorate, Addis Ababa, Ethiopia; 3 Saint Peter’s Specialized Hospital, Research and Evidence Generation Directorate, Addis Ababa, Ethiopia

**Keywords:** Outcomes research, Tuberculosis

## Abstract

Treatment interruption is one of the main risk factors of poor treatment outcome and occurrence of additional drug resistant tuberculosis. This study is a national retrospective cohort study with 10 years follow up period in MDR-TB patients in Ethiopia. We included 204 patients who had missed the treatment at least for one day over the course of the treatment (exposed group) and 203 patients who had never interrupted the treatment (unexposed group). We categorized treatment outcome into successful (cured or completed) and unsuccessful (lost to follow up, failed or died). We described treatment interruption by the length of time between interruptions, time to first interruption, total number of interruption episodes and percent of missed doses. We used Poisson regression model with robust standard error to determine the association between treatment interruption and outcome. 82% of the patients interrupted the treatment in the first six month of treatment period, and considerable proportion of patients demonstrated long intervals between two consecutive interruptions. Treatment interruption was significantly associated with unsuccessful treatment outcome (Adjusted Risk Ratio (ARR) = 1.9; 95% CI (1.4–2.6)). Early identification of patients at high risk of interruption is vital in improving successful treatment outcome.

## Introduction

Treatment adherence is a key component of successful tuberculosis (TB) control programmes and it is an emphases of international and national TB control guidelines^1,2^. However, an estimated 50% of patients on long-term therapy for chronic diseases including TB are non-adherent^3^. TB treatment interruption can occur for short period, which is called intermittent interruption or for greater than two consecutive months that refers to lost to follow up^1,4^. Both intermittent treatment interruption and lost to follow up are the most challenges of TB control programme due to their high risk to severe illness, death, disease transmission, poor treatment outcome and occurrence of drug resistance^5^. All of these health related consequences of treatment interruption have also economic impact in terms of cost and loss of income for patients and their families and cost to the health system^6–8^.

Although multidrug resistant tuberculosis (MDR-TB) treatment considerably save the life of millions^9–11^ and reduce transmission of the resistance strains, significant proportion of patients are interrupting the treatment during the follow up period^4,12–17^. Several interventions including directly observed short course therapy-plus (DOTS-plus) have been also implemented to promote treatment adherence and increase treatment success in MDR-TB patients^18^. However, the proportion of patients who interrupt their treatment remains high^4,16,17,19^. For example, 93% of MDR-TB patients interrupted the treatment at least for one day during follow up period in Philippines^4^, and 20% of MDR-TB patients are non-adherent in Armenia and Abkhazia^12^. Moreover, a meta-analysis recently published showed that 20% of MDR-TB patients are non-adherent^17^.

The occurrence of extensively drug resistant TB (XDR-TB) and totally drug resistant TB (TDR-TB) strains among MDR-TB patients are a major global public health problem^16,20–25^ due to the contagious nature of the disease, difficulty in diagnosis and severe limitation it impose on the options of effective treatment. Among several factors, MDR-TB treatment interruption is the major risk factor for the occurrence of additional drug resistance strains such as XDR-TB or pre–XDR-TB and TDR-TB^20–23^. For instance, MDR-TB patients who lost to follow up significantly developed XDR-TB or pre–XDR-TB^16,20–23^. The risk of poor treatment outcome is also 3–4 times high among patients who intermittently interrupt the treatment for longer periods with sporadic intervals^4^. In contrast, study indicated that the effect of non-adherence is minimal on the occurrence of MDR-TB in TB patients on first line treatment^26^.

Several studies have reported the magnitude of both intermittent treatment interruption and lost to follow up among patients on first line TB treatment^5,27–29^. However, there are limited evidence^4,12,13^ on intermittent treatment interruption, and its effects on treatment outcome in patients with MDR-TB.

Ethiopia is one of the 30 high MDR-TB prevalent countries and the burden of MDR-TB is increasing with an estimated incidence of 2.7% in new and 14% in previously treated cases^30^. Moreover, recently reported meta-analysis in Ethiopia shows that 2.2% of new and 21.1% of previously treated TB cases developed MDR-TB^31^. In Ethiopia, substantial proportion of MDR-TB patients are experienced unsuccessful treatment outcome (lost to follow up, treatment failure and death) which ranges from 21.4 to 35.5%^11,32,33^. However, there is no report from Ethiopia on the magnitude of intermittent treatment interruption and its contribution on unsuccessful treatment outcome in MDR-TB patients. We therefore aimed to determine the distribution of intermittent treatment interruption and its effect on final treatment outcome in MDR-TB patients in Ethiopia. To our knowledge, this study is the first report that presents the intermittent treatment interruption and its effect on treatment outcome in MDR-TB patients in Ethiopia. Hence, determining the magnitude of intermittent treatment interruption and its effect on final treatment outcome in MDR-TB patients are essential to achieve targeted treatment success and prevent XDR and TDR tuberculosis.

## Results

### Participants’ characteristics

**Figure 1:**
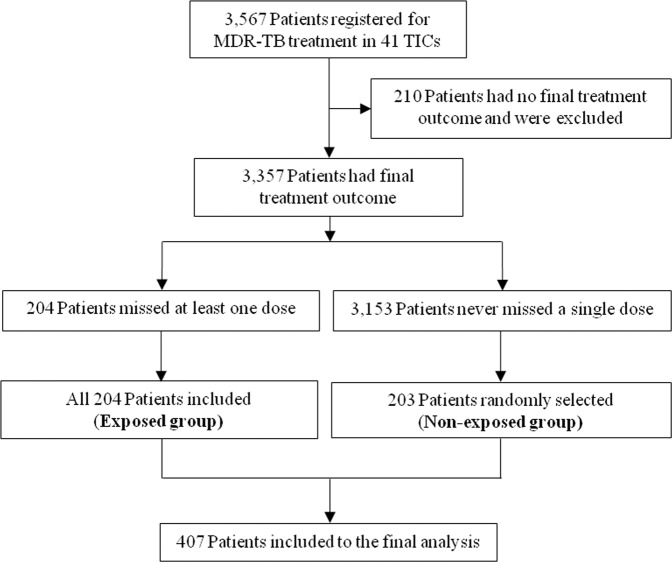
Patient inclusion flow diagram.

A total of 3,357 patients were on MDR-TB treatment and completed their treatment follow up by February 30, 2019 in 41 treatment initiating centres. Of these, 204 patients interrupted the treatment at least for one day (exposed group) and all these patients were enrolled in this study. Of the remaining 3,153 patients who never interrupted the treatment, 203 were selected randomly (unexposed group). Hence, we included a total of 407 MDR-TB patients to this study (Fig. 1).

**Figure 2:**
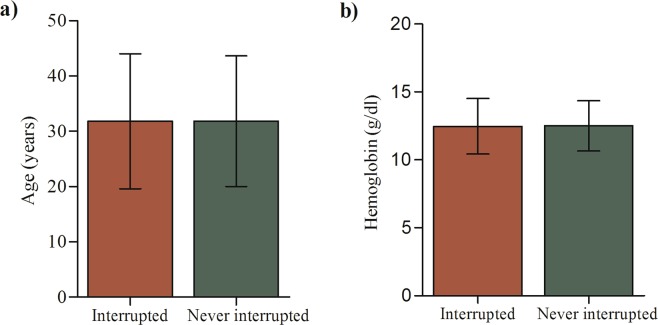
(**a**) Age and (**b**) haemoglobin distributions between interrupted and never interrupted patients at the diagnosis period (Error bar indicated mean ± SD).

Of the total 407 patients, 229 (56.3%) were male and the mean age was 31.8 (±12.0) years with age range of 15 to 80 years (Fig. 2a). 70.3% of the patients had rifampin resistant TB bacilli , and 95.3% patients had pulmonary TB (Table 1). 85.3% of the patients had at least one previous history of TB treatment prior to current MDR-TB treatment, and 17 (4.2%) had previous exposure to second-line drugs (Table 1). 383 (94.1%) of the patients were bacteriologically diagnosed to enter into the treatment, and 306 (75.2%) of the patients diagnosed by geneXpert (Table 1). 20.6% of the patients were infected with human immunodeficiency virus (HIV) (Table 1) and of those HIV infected patients 86.9% (73/84) were on ART. Regarding patients’ characteristics at treatment initiation, there was no significant difference between patients who interrupted the treatment at least for one day and those who never interrupted during the treatment period (Table 1, Fig. 2).

**Table 1:** Participants’ characteristics.

Variable		At least one day interrupted [n = 204] Frequency%	Never interrupted [n = 203] Frequency%	P-value	Total n(% = n/407 × 100)
Sex	Male	108 (47.2%)	121 (52.8%)	0.175	229 (56.3)
	Female	96 (53.9)	82 (46.1)		178 (43.7)
Age in year	15–24	62 (55.9)	49 (44.1)	0.305	111 (27.3)
	25–35	88 (46.3)	102 (53.7)		190 (46.7)
	36–50	29 (56.8)	33 (53.2)		62 (15.2)
	≥50	25 (56.8)	19 (43.2)		44 (10.8)
Drug resistance type	Rifampin resistant/Isoniazid susceptibility status unknown	143 (50.0)	143 (50.0)	0.996	286 (70.3)
	Multidrug resistant	49 (50.5)	48 (49.5)		97 (23.8)
	Unknown	12 (50.0)	12 (50.0)		24 (5.9)
Resistance diagnosis method	Xpert	151 (49.3)	155 (50.7)	0.892	306 (75.2)
	Culture	41 (53.2)	36 (46.8)		77 (18.9)
	Clinical	12 (50.0)	12 (50.0)		24 (5.9)
Anatomical site of the disease	Pulmonary	193 (49.7)	195 (50.3)	0.488	388 (95.3)
	Extra pulmonary	11 (57.9)	8 (42.1)		19 (4.7)
Previous treatment history	New	26 (43.3)	34 (56.7)	0.255	60 (14.7)
	Previously treated	178 (51.3)	169 (48.7)		347 (85.3)
Previous history of SLDs exposure	Yes	10 (58.8)	7 (41.2)	0.483	17 (4.2)
	No	194 (50.1)	193 (49.9)		387 (95.1)
Reasons for entering to MDR-TB treatment	Bacteriologically diagnosed	192 (50.1)	191 (49.9)	0.990	383 (94.1)
	Clinically diagnosed	12 (50.0)	12 (50.0)		24 (5.9)
HIV sero-status	Non-reactive	160 (50.5)	157 (49.5)	0.939	317 (77.9)
	Sero-reactive	42 (50.0)	42 (50.0)		84 (20.6)
	Not recorded	2 (1.0)	4 (2.0)		6 (1.5)
ART status	Not applicable	160 (50.5)	157 (49.5)	0.858	317 (77.9)
	ART started	36 (49.3)	37(50.7)		73 (17.9)
	Not recorded	8 (3.6)	9 (4.4)		17 (4.2)

MDR- multidrug resistant, TB-tuberculosis, SLDs- second line drugs, HIV-human immunodeficiency virus, ART-antiretroviral therapy.

### Treatment interruption

A total of 3,357 patients were registered for MDR-TB treatment and had final treatment outcome during the study period; of these 204 patients missed at least a single dose of treatment over the course of treatment period, but for less than two consecutive months. Thus, the overall proportion of intermittent treatment interruption was 6.1% (204/3,357). Table 2 depicts the distribution of treatment interruption over the period of the treatment. The median time on treatment among patients who interrupted at least one dose was 19.8 (IQR, 8.0–22.0) months, while the median of total number of missed doses was 12.5 (IQR, 5.0–24.0) days. Furthermore, the median time to first interruption was 74.5 (IQR, 31.0–135.5) days. The total median episode of treatment interruption was 2.0 (IQR, 2.0–4.0) episodes, and the maximum interruption episode was 20 episodes. The median proportion of total number of treatment missed was 2.7% (IQR, 1.1%–10.0%) over the treatment period. More than half of the patients (112/204; 54.9%) missed more than 10 doses of their treatment.

**Table 2:** Treatment interruption characteristics in MDR-TB patients who missed at least one dose during the treatment duration (n = 204).

Characteristic	Median (IQR)	Range
Total days on treatment (in months)	19.4 (7.6–21.6)	0.17–34.8
Total number of missed doses (in days)	12.5 (5.0–24.0)	1.0–119
Time to first interruption (in days)	74.5 (31.0–135.5)	2.0–710
Percentage of total missed doses (in %)	2.7 (1.1–10.0)	0.2–100
Total number of interruption episode (number)	2.0 (1.0–4.0)	1.0–20

IQR- Inter-quartile range.

**Figure 3:**
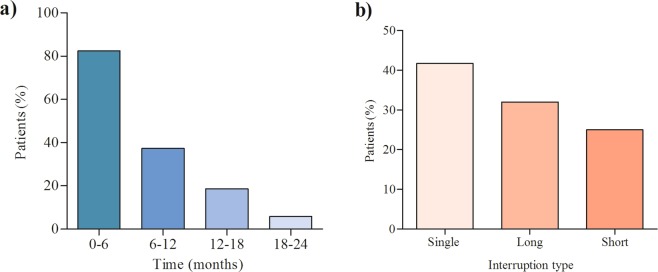
(**a**) Treatment interruption distribution in six month categories of overall treatment period; (**b**) Interruption type and length of interruption distribution between consecutive treatment interruption episodes.

Eighty two percent of the patients interrupted their treatment within 6 months of treatment initiation (Fig. 3a). About 40% of the patients interrupted the treatment only once over the course of the treatment whereas more than half of the patients interrupted the treatment more than one times (episodes) (Fig. 3a). 32.8% of the patients demonstrated long interval between two consecutive interruptions, while 25.5% were short interval (Fig. 3b). We conducted a Univariate Poisson regression with robust standard error to assess the differences between single, short and long interval interruptions on treatment outcome within the patients who interrupted the treatment for at least one day. There was a significant difference between single and short interruption interval on treatment outcome (p < 0.001); between single and long interruption interval (p = 0.003) and between short and long interruption intervals (p < 0.001).

### Treatment outcome

Of 407 patients included to this study 222 (54.5%) were cured, 71 (17.4%) were lost to follow up, 55 (13.5%) completed the treatment, 50 (12.3%) died and treatment of 9 (2.2%) patients failed. The overall treatment success (cured plus treatment completed) was 277 (68%), and the overall unsuccessful treatment outcome (lost to follow up, treatment failed and death) was 130 (31.9%).

**Figure 4:**
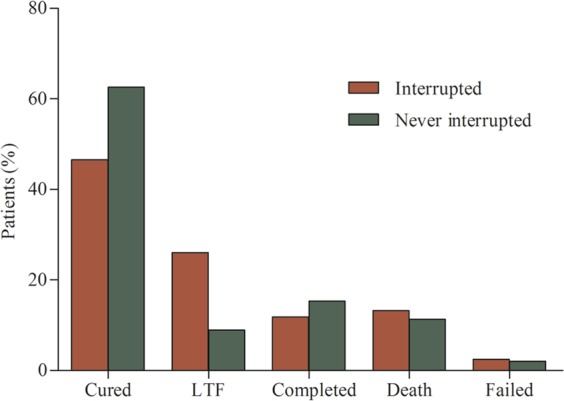
Treatment outcome proportion between patients interrupted at least one dose and not interrupted the treatment (LFT- Lost to follow up, p-value for cured <0.001, lost to follow up <0.001).

Of 203 patients who never interrupted the treatment 127 (62.6%) were cured, and of 204 patients who interrupted the treatment at least once during treatment period 95 (46.6%) were cured (62.6% vs 46.6%; p < 0.001) (Fig. 4). Of patients who interrupted the treatment at least one dose over the course of the treatment, 24 (11.8%) completed the treatment, while 31 (15.3%) were complete from those who never interrupted the treatment (Fig. 4). Lost to follow up was nearly three times higher in the patients who interrupted the treatment at least once, than those who never interrupted (26% vs 8.9%; p < 0.001) (Fig. 4).

In Univariate Poisson regression model with robust standard error treatment interruption at least for one day (Unadjusted risk ratio (URR) = 1.9; 95% CI (1.4–2.5)), HIV sero-reactive (URR = 1.5; 95% CI (1.1–2.0)), pulmonary TB type (URR = 0.59; 95% CI (0.3–0.92)), being on ART (URR = 1.5; 95% CI (1.1–2.0)) and presence of any grade of anaemia (UOR = 1.5; 95% CI (1.1–1.9)) were significantly associated with poor treatment outcome (Table 3). In multivariable Poisson regression model with robust standard error which adjusted for age, previous TB treatment history, anatomical site of TB (pulmonary versus extra-pulmonary), HIV sero-status and anaemia, treatment interruption at least for one day was significantly associated with unsuccessful treatment outcome (ARR = 1.9; 95% CI (1.4–2.6), P < 0.001) (Table 3). In addition, HIV sero-status (ARR = 1.4; 95% CI (1.1–1.9), P = 0.016) and anaemia (ARR = 1.3; 95% CI (1.03–1.8), P = 0.049) were significantly associated with unsuccessful treatment outcome (Table 3). ART status was not included to the multivariable model because of strong co-linearity it shown with HIV sero- status.

**Table 3:** The effect of treatment interruption and other factors on unsuccessful treatment outcome in MDR-TB patients.

Variable		Treatment outcome		Unadjusted model		Adjusted model	
		Successful, n (%)	Unsuccessful, n (%)	URR(95% CI)	P-value	ARR(95%CI)	P-Value
Treatment interruption status	Never interrupted	158 (77.8)	45 (22.2)	1.00		1.00	
	At least one day interrupted	119 (58.3)	85 (41.7)	1.9 (1.4–2.5)	< 0.001*	1.9 (1.4–2.6)	< 0.001*
Sex	Male	156(68.1)	73 (31.9)	1.00			
	Female	121 (68.0)	57(32.0)	1.0 (0.75–1.3)	0.975		
Age in year				1.1 (0.99–1.1)	0.091	1.0 (0.99–1.1)	0.203
Resistance type	Rifampin resistant/Isoniazid susceptibility unknown	194 (66.2)	99 (33.8)	1.00			
	Multidrug resistant	83 (72.8)	31 (27.2)	0.80 (0.57–1.1)	0.212		
Anatomical site of TB	Extra-pulmonary	9 (47.4)	10 (52.60	1.00		1.00	
	Pulmonary	268 (69.10	120 (30.9)	0.59 (0.37–0.92)	0.021	0.64 (0.40–1.04)	0.072
Previous treatment history	New	36 (60.0)	24 (40.0)	1.00		1.00	
	Previously treated	241 (69.5)	106 (30.5)	0.76 (0.54–1.1)	0.130	0.83 (0.59–1.2)	0.273
Previous history of SLDs exposure	No	263 (68.0)	124 (32.0)	1.00			
	Yes	11 (64.7)	6 (35.3)	1.1 (0.57–2.1)	0.774		
Reasons for entering to MDR-TB treatment	Bacteriologically diagnosed	263 ()68.7	120 (31.3)	1.00			
	Clinically diagnosed	14 (58.3)	10 (41.7)	1.3 (0.81–2.2)	0.261		
HIV sero-status	Non-reactive	225 (71.0)	92 (29.0)	1.00		1.00	
	Sero-reactive	48 (57.1)	36 (42.9)	1.5 (1.1–2.0)	0.011*	1.4 (1.1–1.9)	0.016*
ART status	Not applicable	225 ()71.0	92 (29.0)	1.00			
	ART started	42 (57.5)	31 (42.5)	1.5 (1.1–2.0)	0.019*		
Anaemia	Normal	155 (73.8)	55 (26.2)	1.00		1.00	
	Any grade of anaemia present	122 (61.9)	75 (38.1)	1.4 (1.1–1.9)	0.011*	1.3 (1.03–1.8)	0.049*

MDR-multidrug resistant, TB-tuberculosis, SLDs- second line drugs, URR-unadjusted risk ratio, ARR-Adjusted risk ratio, CI-Confidence interval, HIV-Human immunodeficiency virus, ART-antiretroviral therapy, *-statistically significant variable at p-value 0.05.

### Multidrug resistance status at treatment initiation

Table 4 depicts the distribution of drug resistance status by treatment outcome and treatment interruption. Of the total of 407 patients, 383 (94.1%) had drug susceptibility test for rifampin at treatment initiation. Of these, 263 (68.7%) were treated successfully and 192 (50.1%) interrupted the treatment at least for one day (Table 4). In the same manner, of the total of 407 patients, 107 (26.3%) had drug susceptibility test for isoniazid at the treatment initiation. Of these, 69 (73.4%) were treated successfully and 51 (54.3%) interrupted the treatment at least for one day (Table 4).

**Table 4:** Drug resistance distribution by treatment outcome and treatment interruption groups.

Drug and its susceptibility status		Treatment outcome status, n (%)		Treatment interruption status, n (%)	
		Successful	Unsuccessful	Interrupted at least one day	Never interrupted
Rifampin (n = 383)	Resistant	263 (68.7)	120 (31.3)	192 (50.1)	191 (49.9)
	Susceptible	Not reported	Not reported	12 (50.0)	12 (50.0)
Isoniazid (n = 107)	Resistant	69 (73.4)	25 (26.6)	51 (54.3)	43 (45.7)
	Susceptible	6 (46.2)	7 (53.8)	10 (76.9)	3 (23.1)
Ethambutol (n = 18)	Resistant	7 (77.8)	2 (22.2)	6 (66.7)	3 (33.3)
	Susceptible	3 (33.3)	6(66.7)	7 (77.8)	2 (22.2)
Streptomycin (n = 16)	Resistant	7 (63.6)	4 (36.4)	7 (63.6)	4 (36.4)
	Susceptible	2 (40.0)	3 (60.0)	5 (100.0)	0 (0.0)

## Discussion

The occurrence of additional drug resistant (XDR and TDR) TB is increasing across the world^20–25^ and severely limiting the options of effective treatments. Treatment interruption is one of the main risk factor for the occurrence of additional drug resistance in MDR-TB patients^4,12,13^. However, few published evidence tried to report the magnitude of intermittent treatment interruption and its effect on final treatment outcome in MDR-TB patients^4,12,13^. Thus, we attempted to determine the magnitude of intermittent treatment interruption and its effect on final treatment outcome in MDR-TB patients in Ethiopia.

The proportion of intermittent treatment interruption in MDR-TB patients in Ethiopia in last ten years was 6.1%. The majority of these patients interrupted the treatment in the first six month of the treatment period. Moreover, the majority of the patients demonstrated long intervals between two consecutive interruptions, and the risk of poor treatment outcome was 1.9 times higher in the patients who interrupted the treatment at least one day than those who never interrupted. Lost to follow up was nearly three times (26%) in the patients interrupted the treatment at least once, than those who never interrupted (8.9%). HIV sero-status and presence of any grade of anaemia were also significantly associated with poor treatment outcome.

Previous study reported that 93% of MDR-TB patients have interrupted the treatment at least once during the treatment period^4^. This finding is considerably larger than our finding in which only 6.1% of the patients interrupted the treatment at least once during the follow up period. This difference most probably happened due to adherence registration, programme monitoring quality, treatment follow up modality and study participant differences. In our study, adherence status of several patients was not recorded on treatment card category IV and their adherence status was unknown. This might be underestimated the proportion of intermittent treatment interruption in our study.

In the current study, the majority of the patients interrupted the treatment in the first six month of the treatment period. This finding is similar with the previous study in which the majority of patients interrupted the treatment in the early stage of the treatment period^4^. Previous study on drug susceptible TB patients indicated that TB patients more likely interrupt the treatment after the intensive phase of the treatment as the result of symptoms related to the disease resolved or the patient felt better^34^. However, this study^34^ result was in contrast to our finding in which the majority (82%) of the patients interrupted at early period (0 to 6 months) of the treatment. Interruptions at early stage of treatment before the full clearance of the bacteria could be critical due to the high risk of potential replication of the resistant bacteria and the occurrence of additional drug resistance strains. This early interruption of the treatment is due to severe adverse drug reactions associated with MDR-TB drugs^12^. The majority of the patients demonstrated long interval between two consecutive interruptions in this study. This finding was similar with the previous studies in which the interval between two consecutive interruptions was long for considerable proportion of patients^4,12^.

Missing treatment at least for one day was significantly associated with poor treatment outcome among MDR-TB patients in the current study. Our finding is consistent with the previous study in which the risk of treatment interruption was 3–4 times higher in patients who interrupted the treatment for long interval^4^. Similarly, study reported by Bastard *et al*.^12^ has shown significant association between intermittent treatment interruption and the occurrence of second line drugs (SLDs) resistance. This finding is in agreement with our finding in which treatment interruption was significantly associated with unsuccessful treatment outcome. Furthermore, the occurrence of additional resistance is most likely high in the treatment failed patients. Poor treatment outcome might happen due to the occurrence of drug resistance as a result of treatment interruption^12^. Another previous study has also indicated a significant association between adherence less than 80% and the development of XDR-TB in MDR-TB patients^23^. This is programmatically and economically important because several studies have shown that the acquisition of additional resistance is increasing across the world and is significantly associated with poor treatment outcome^12,13,16,20–23^. This may severely limit the option of effective treatment and could impact the global economy. We could not find any study that shows the effect of intermittent treatment interruption on lost to follow in patients on MDR-TB treatment. However, a previous study on drug-susceptible TB patients has shown a significant effect of intermittent treatment interruption on lost to follow up^27^. The finding of this study on drug-susceptible TB patients affirms our finding on MDR-TB patients in which lost to follow up was three times higher in the patients who missed the treatment at least for one day.

In the current study, HIV sero-status and presence of any grade of anaemia were also significantly associated with poor treatment outcome. This study finding was similar with the previous studies^11,15,32^ in which HIV sero-reactive significantly associated with poor treatment outcome in MDR-TB patients. Similarly, previous study indicated that the presence of anaemia is associated with poor treatment outcome^32^. This finding was similar with our finding in which the risk of poor treatment outcome was 1.6 times higher in the patients who had any grade of anaemia than those who have no anaemia.

Our study has limitations; the main one is its retrospective study design in which the data was extracted from routine MDR-TB programme registration that lacks sociodemographic, behavioural, adverse drug reactions and key laboratory variables. These limited our study to assess factors associated with treatment interruption which could be important for TB control programme. In addition, lack of key variables limited our ability to further explore risk factors of poor treatment outcome. Thus, the risk factors of poor treatment outcome may not be limited to treatment interruption, HIV sero-reactivity and the presence of anaemia. In addition, lack of key variables could have resulted in an underestimation of the effect of different variables in the model. Prospective studies that use validated data collection instruments required to determine factors associated with treatment interruption and poor treatment outcome in MDR-TB patients. Another limitation of this stud was treatment adherence status of several patients was not recorded in category IV treatment card. This might have underestimated or overestimated the proportion of intermittent treatment interruption which could also underestimate the effect of treatment interruption on the final treatment outcome.

## Conclusion

Our study create concept for the future studies on the intermittent treatment interruption and its effect on the poor treatment outcome in MDR-TB patients which has impact on TB control programme. In the current study the majority of the patients interrupted the treatment in the first six month of treatment period which could lead to disease transmission and additional resistance acquisition. Intermittent treatment interruption was significantly associated with unsuccessful treatment outcome. Early identification of patients at high risk of interruption through careful adherence monitoring is vital in preventing treatment interruption and improving successful treatment outcome. Future prospective cohort study is required to identify the underlying reasons of intermittent interruption and its effect on final treatment outcome.

## Methods

### Study setting and design

We conducted a retrospective cohort study in patients ≥15 years old and those who were treated for MDR-TB in past 10 years (February 2009 to February 2019) at 41 MDR-TB Treatment Initiating Centres (TICs) in Ethiopia. A total of 3,357 patients completed their MDR-TB treatment follow up and had their final treatment outcome by February 30, 2019 in 41 treatment initiating centres. Of these, 204 patients interrupted the treatment at least for one day but for less than two consecutive months (exposed group) and all these patients were enrolled in the study (Fig. 1). Of the remaining 3,153 patients who never interrupted the treatment, 203 were selected by simple random sampling techniques using registration book as sampling frame from the same TICs where the interrupted patient sampled (unexposed group) (Fig. 1). Overall, we included a total of 407 MDR-TB patients to this study (Fig. 1).

Programmatic management of MDR-TB was started in single hospital by February 2009 in Ethiopia^11^. Currently, MDR-TB TICs are decentralized to different levels of health care system in the country reaching 53 TICs during this study period and several treatment follow up centres (TFCs). The majority of MDR-TB patients initiate their treatments in TICs while clinically stable patients follow their scheduled second line drugs (SLDs) under DOTS programme in TFCs^2^. Although clinically stable patients follow their treatment at TFCs, all information on the patients enrolled into the MDR-TB treatment has been documented at TICs. Of the total of 53 TICs, 41 had patients who have final treatment outcome during the study period (Fig. 1). Of these we included 30 TICs (73.2%) that have recorded intermittent treatment interruption on at least one patient.

### Inclusion and exclusion criteria

We included patients who were ≥15 years old, rifampin resistant, multidrug resistant and diagnosed either clinically or bacteriologically. In this study, clinically diagnosed MDR-TB refers to those cases with no documented drug susceptibility test (DST) results but treated empirically with a course of treatment including SLDs based on clinical criteria and contact history^2^. Whereas, bacteriologically confirmed MDR-TB refers to those cases with documented DST results. In addition, we included only patients treated under national TB programme by a standardized long regimen type of treatment in 30 selected TICs from February 2009 to February 2019 and had final treatment outcome. However, we excluded patients whose final treatment outcome was unknown. Patients who transferred from other TICs and TFCs after taking the treatment for more than one month were also excluded because all the information regarding the patients is recorded in the previous TIC according to national treatment guideline^2^. We were also excluded patients who completed the follow up period and whose final treatment results not evaluated.

### Diagnosis of multidrug resistant tuberculosis

In Ethiopia, drug susceptibility test was done by LJ (Löwenstien-Jensen) and BACTEC MGIT 960 phenotypic methods and MTBDRplus/MTBDRsl LPA (Line Probe Assay) and Xpert MTB/RIF assay genotypic methods. *Mycobacterium tuberculosis* was isolated by inoculating decontaminated sputum sample into LJ and MGIT 960 culture media. For the isolation of extra pulmonary TB, body fluids such as pleural fluid, cerebrospinal fluid, lymph node aspirates were taken from any part of the body where the disease manifested and inoculated into both LJ and MGIT culture media.

### Multidrug resistant tuberculosis treatment

MDR-TB patients were treated based on the national treatment guideline which was developed from national and international evidence and recommendations^2^. Previously, all MDR-TB patients were treated as inpatient model of care for the first few months until the patient become stable at treatment centers in Ethiopia. However, according to the recent edition of national TB guideline, all patients with MDR-TB need to be treated under clinic-based ambulatory model of care, unless the patient clinically unstable or developed sever adverse drug reaction during the course of treatment^2^. Patients either with serious medical or social conditions could be admitted with the decision of the treatment panel^2^. The current MDR-TB treatment drugs under long regimen in Ethiopia consists SLDs: levofloxacin, ethionamide, cycloserine, para-aminosalicyclic acid (PAS), pyrazinamide, prothionamide, linezolid, clofazimine and injectable drugs such as amikacin, kanamycin and capreomycin^2^. Treatment with injectable drugs continues at least for eight months based on clinical, microbiological and radiographic evaluation outcomes^2^. The minimum treatment duration was 20 months for long regimen which is at least 18 months after bacteriological conversion^2^.

Ethiopia has provided standardized long treatment regimen for all patients diagnosed for MDR-TB^2^. However, since a global recommendation on short regimen by WHO in 2016, Ethiopia has introduced the standardized short treatment regimen as the preferred regimen for the treatment of MDR-TB patients in addition to the long regimen^2^. The maximum duration of the short treatment regimen is nine to 11 months^2^ which benefits the patients and the health system as it significantly decreases the treatmentduration^2^. In Ethiopia, the short treatment regimen consists of the following SLDs: kanamycin, amikacin, moxifloxacillin, clofazimine, pyrazinamide, ethambutol and high-dose isoniazid^2^.

Laboratory tests, chest X-ray and clinical investigations are used to monitor response to the treatment and to identify treatment related complications in patients on MDR-TB treatment in Ethiopia^2^. For extra-pulmonary TB, clinical investigations are only used to monitor response to the treatment, while laboratory tests are used to identify treatment related complications.

### Data collection

We collected data through reviewing clinical charts, registration books and laboratory reports on socio-demographic variables such as sex, age and regional state. We also collected data on clinical characteristics including anatomical site of TB (pulmonary versus extra pulmonary), drug resistance type, previous treatment history, diagnosis method, HIV sero-status and Antiretroviral Therapy (ART) status. In addition, we collected information on bacteriological status (smear, Xpert MTB/RIF, culture or DST results) during the diagnosis of TB. All data were collected by health professionals familiar with MDR-TB treatment after two days of practical training on data collection tool.

### Exposure and outcome variables definition

The outcome variable in this study was final MDR-TB treatment outcome, and the exposure variable was intermittent treatment interruption. In this study, intermittent treatment interruption refers any time a patient missed a prescribed dose of treatment for at least one day within two consecutive months based on previous study^4^. The final MDR-TB treatment outcome in this study was directly adapted from WHO and national MDR-TB treatment guidelines definitions^1,2^. The final treatment outcome categories of MDR-TB were cured, treatment completed, treatment failed, lost to follow up and death. The definitions of these MDR-TB treatment outcome categories are given as follows based on WHO and national treatment guidelines^1,2^. Cured is defined as a patient initially bacteriologically confirmed and completed the treatment without the evidence of treatment failure and three or more consecutive cultures taken at least 30 days apart are negative after the intensive phase. Treatment completed is defined as a patient who completed the treatment without the evidence of treatment failure but there is no record that indicates three or more consecutive cultures taken at least 30 days apart are negative after the intensive phase. A patient whose treatment is terminated or need for permanent regimen change of at least two anti-TB drugs is categorized as treatment failure. Moreover, lost to follow up refers to a patient whose treatment is interrupted for two consecutive months or more.

In this analysis, we categorized the treatment outcome in to two categories: successful (cured and treatment completed) and unsuccessful (death, treatment failed and lost to follow up). Data on interruption status was extracted from category IV MDR-TB patient’s treatment card. Information on final treatment outcome was collected from TB registration book and category IV MDR-TB patient’s treatment card.

### Data analysis

We entered data into CSPro software version 6.1 at data collection sites and transferred to Microsoft Excel for cleaning. All data was analyzed by STATA version 14 (StataCorp, College Station, TX, USA). Before main analysis, all data were confirmed and cleaned from each data source. We described participants’ demographic and clinical characteristics using descriptive statistics, and compared patients’ characteristics between patients who interrupted the treatment at least one dose (considered as exposed) and patients who never interrupted the treatment (considered as unexposed) over the course of the treatment duration. We also calculated the overall proportion of intermittent treatment interruption by dividing the number of patients who interrupted the treatment at least for one day to the total number of patients registered for MDR-TB treatment in the country and had final treatment during the study period.

We described the treatment interruption in detail by the median number of date the patients on the treatment, median number of missed doses, median date of time to first interruption, median percentage of missed doses and median number of interruption episode. The percentage of missed doses was calculated by dividing the total number of interrupted date to the total number of date the patient on the treatment which multiplied by 100%. We also described the treatment interruption by the length of date between two consecutive interruptions (time on treatment between two interruptions). We categorized the length of date between two consecutive interruptions by using the pooled median of time on treatment across whole interruption episodes. As a result, patients who were on treatment for less than the pooled median (<2.5 days) on the treatment were categorized under short interval, while those were on the treatment for ≥ pooled median (≥2.5 days) categorized under long interval. The total number of interruption episode is defined as the total number of interruption times that the patient interrupted the treatment over the course of the treatment.

We fit Poisson regression model with robust standard error to assess the effect of treatment interruption on the final treatment outcome, and the association between different interruption intervals length. Patients who interrupted the treatment at least for one day were considered as exposed group, whereas those never interrupted as unexposed group in this analysis (Fig. 1). We calculated unadjusted and adjusted risk ratios (URR and ARR) with 95% Confidence Intervals (CIs) to determine association between treatment interruption and treatment outcome. To estimate the independent effect of treatment interruption on the final MDR-TB treatment outcome, we adjusted for the variables scored p-value ≤ 0.2 during simple Poisson regression analysis.

We categorized haemoglobin level into two categories (normal versus any grade of anaemia present) based on WHO anaemia categorization cut-off points for adults^35^. Since the missing value was not more than 5% of the total patients, we input the mean of haemoglobin for the missed value. We set level of significance at 5% for all analysis, and two tailed test was employed.

### Ethical consideration

This study was approved by the ethics review boards of Tehran University of Medical Sciences (IR.TUMS.SPH.REC.1396.4287), Ethiopian Public Health Institute (EPHI-IRB-065–2017), Saint Peter’s Specialized Hospital (V81622018) and Armouer Hansen Research Institute (PO13/18). All methods used in this study were also performed according to relevant guidelines and regulation of biomedical researches; and waiver of informed consent was obtained from each review board.

## Data Availability

The datasets generated during and/or analysed during the current study are available from the corresponding author on reasonable request.
